# Retraction of “miR-654-5p promotes gastric cancer progression via the GPRIN1/NF-κB pathway”

**DOI:** 10.1515/med-2025-9995

**Published:** 2025-04-02

**Authors:** Weidong Zhou, Peifei Li, Peihua Jin

**Affiliations:** Department of Gastroenterology, Hwa Mei Hospital, University of Chinese Academy of Sciences (Ningbo No. 2 Hospital), 41 Xibei Street, Ningbo 315010, Zhejiang, China; Department of Gastroenterology, Ningbo First Hospital, Ningbo 315010, Zhejiang, China; Department of Gastroenterology, Hwa Mei Hospital, University of Chinese Academy of Sciences (Ningbo No. 2 Hospital), Ningbo 315010, Zhejiang, China

Retraction of: Zhou W, Li P, Jin P. MiR-654-5p promotes gastric cancer progression via the GPRIN1/NF-κB pathway. Open Medicine 2021;16(1):1683–1695, DOI: 10.1515/med-2021-0369

This article has been retracted by agreement between the Editor-in-Chief, Professor Vittorio Calabrese, and the publisher, Walter De Gruyter GMBH, following a thorough investigation. The retraction is due to misleading usage of the figures and undisclosed research outsourcing. Figures 2c, 2d, 2e, and 4b, 4c, 4d were wrongly included in the article and they are not connected to results. In addition, all presented experiments have been conducted by an unspecified external company, which raises questions about the integrity and rigor of the published research.

